# Comparison of Multiplexed Immunofluorescence Imaging to Chromogenic Immunohistochemistry of Skin Biomarkers in Response to Monkeypox Virus Infection

**DOI:** 10.3390/v12080787

**Published:** 2020-07-23

**Authors:** Anup Sood, Yunxia Sui, Elizabeth McDonough, Alberto Santamaría-Pang, Yousef Al-Kofahi, Zhengyu Pang, Peter B. Jahrling, Jens H. Kuhn, Fiona Ginty

**Affiliations:** 1 GE Research, 1 Research Circle, Niskayuna, NY 12309, USA; Anup.Sood@ge.com (A.S.); yunxia.sui@gmail.com (Y.S.); elizabeth.mcdonough@ge.com (E.M.); santamar@ge.com (A.S.-P.); alkofahi@ge.com (Y.A.-K.); pang@ge.com (Z.P.); 2 Integrated Research Facility at Fort Detrick, National Institute of Allergy and Infectious Diseases, National Institutes of Health, B-8200 Research Plaza, Frederick, MD 21702, USA; jahrlingp@niaid.nih.gov

**Keywords:** immunohistochemistry, IHC, monkeypox, monkeypox virus, MPXV, multiplexed immunofluorescence, MxIF, *Orthopoxvirus*, *Poxviridae*, poxvirus

## Abstract

Over the last 15 years, advances in immunofluorescence-imaging based cycling methods, antibody conjugation methods, and automated image processing have facilitated the development of a high-resolution, multiplexed tissue immunofluorescence (MxIF) method with single cell-level quantitation termed Cell DIVE^TM^. Originally developed for fixed oncology samples, here it was evaluated in highly fixed (up to 30 days), archived monkeypox virus-induced inflammatory skin lesions from a retrospective study in 11 rhesus monkeys to determine whether MxIF was comparable to manual H-scoring of chromogenic stains. Six protein markers related to immune and cellular response (CD68, CD3, Hsp70, Hsp90, ERK1/2, ERK1/2 pT202_pY204) were manually quantified (H-scores) by a pathologist from chromogenic IHC double stains on serial sections and compared to MxIF automated single cell quantification of the same markers that were multiplexed on a single tissue section. Overall, there was directional consistency between the H-score and the MxIF results for all markers except phosphorylated ERK1/2 (ERK1/2 pT202_pY204), which showed a decrease in the lesion compared to the adjacent non-lesioned skin by MxIF vs an increase via H-score. Improvements to automated segmentation using machine learning and adding additional cell markers for cell viability are future options for improvement. This method could be useful in infectious disease research as it conserves tissue, provides marker colocalization data on thousands of cells, allowing further cell level data mining as well as a reduction in user bias.

## 1. Introduction

Histochemical scores (H-scores) are the gold-standard semi-quantitative assessment of cellular markers in immunohistochemically (IHC) stained tissues [1,2,3]. This quantitation method produces discrete data, is time-consuming, subject to observer bias and requires serial sections of tissue. However, Cell DIVE, a multiplex immunofluorescence (MxIF) microscopy method allows multiple protein markers to be assessed in a single section of formalin-fixed, paraffin-embedded (FFPE) tissue. It involves iterative staining, imaging and signal inactivation cycles and computational registration of sequential DAPI images [4,5,6]. By repeatedly analyzing the same tissue sample, Cell DIVE conserves tissue, improves consistency, provides marker colocalization data, and due to automated biomarker quantification, potentially reduces bias. In addition, antibody dye labeling is direct, reducing the incidence of nonspecific or background signals and enhancing specificity. To reduce autofluorescence in FFPE tissue using Cell DIVE, autofluorescence is imaged before staining and subsequently subtracted [7,8] from the biomarker signal. Markers specific for different cell and tissue types are included in the staining workflow and this allows cell-level segmentation and quantification of biomarker intensity and separation into different tissue regions, providing thousands of cellular datapoints per field of view.

Cell DIVE has been used extensively for analysis of biomarker colocalization at cell level and immune cell characterization of cancerous lesions, which are typically formalin-fixed for short periods (2–48 h) [4,5,6,9,10,11]. However, it has not yet been evaluated systematically for its potential as a tool to investigate the pathogenesis of high-consequence infectious disease agents, where samples are fixed for long periods to safeguard against accidental infection. It could be particularly useful in studies of viral risk group 3–4 select agents, which can only be handled in biosafety level 3 or 4 biocontainment facilities [12,13]. The relatively low number of these facilities dictates overall low study numbers, and thereby scant availability of animal tissues. Therefore, increasing the number of biomarkers per slide would save scarce/precious tissue and avoid variability associated with analyzing serially sectioned tissue samples and allow assessment of biomarker co-expression.

To determine whether this multiplexed analysis technique could be applied to high-consequence viral pathogens, we compared manually quantified chromogenic IHC (H-scores) to automated quantification of cell biomarker intensity and immune cell counts via Cell DIVE in archived samples of well-characterized tissue lesions from retrospective studies in macaques infected with monkeypox virus (MPXV), a risk group 3 select agent requiring biosafety level 3 containment [12,13]. MPXV is a double-stranded DNA virus (*Poxviridae*: *Chordopoxvirinae*: *Orthopoxvirus*) [14] that causes monkeypox in humans (International classification of diseases [ICD] 10 code B04; ICD-11 code 1E71) [15,16]. MPXV infection is characterized by vesiculopustular skin lesions with marked epidermal hyperplasia, hydropic degeneration and a florid inflammatory cell response that are markedly similar in diverse animal models and humans [17,18,19,20,21,22,23,24], therefore providing a highly defined environment for methodological comparisons.

To compare automated cell biomarker data and H-scores, we selected a variety of nuclear and cytoplasmic markers for quantitation from FFPE skin lesions caused by one of two isolates of MPXV (Table 1). During orthopoxvirus infections, heat-shock protein 70 (Hsp70) concentrations increase in a variety of cell types [25,26,27,28,29], whereas Hsp90 concentrations do not [28,29]. However, VACV infection causes a change in intracytoplasmic localization of Hsp90 inducing an association between this chaperone molecule and virosomes [30]. Consequently, we stained for heat-shock protein markers to determine if we could detect changes in the subcellular localization of these proteins in the MPXV-induced lesions.

Numerous viruses, including orthopoxviruses, use the mitogen activated protein kinase (MAPK)/phosphorylated extracellular signal-regulated kinases 1 and 2 (ERK1/2; phosphorylated at threonine 202 and tyrosine 204, pT202_pY204) pathway [31]. In vitro, CPXV and VACV stimulate this kinase pathway from early to late phases of the virus infection cycle [31,32,33,34,35,36,37]. To determine whether MPXV infection in vivo induces phosphorylation of ERK1/2 in the epidermis and translocation of this protein into the nucleus, we quantified ERK1/2 and ERK1/2_pY204 expression in the nuclei and cytoplasm of epithelial cells in MPXV-lesioned and adjacent epidermal tissues. Cluster of differentiation (CD) markers were also used to quantify inflammation due to infiltrates of macrophages (CD68^+^) and T-cells (CD3^+^). Additional segmentation markers were used to stain the cell membrane (E-cadherin [E-Cad], Na^+^/K^+^-ATPase), cytoplasm (ribosomal protein S6 [RPS6]), nucleus (4′,6′-diamidino-2-phenylindole, dihydrochloride [DAPI]) and epithelium (pan-cytokeratin). These were used to segment all cells in the imaged fields of view and allowed cellular quantification of biomarker intensity and finally comparison with pathologist-generated H-scores.

## 2. Materials and Methods

### 2.1. Sample Selection, Biomarker Selection and Antibody Validation

For this retrospective study (conducted at NIH/NIAID/IRF-Frederick in 2011), archived vesiculopustular skin lesions from 11 rhesus monkeys (*Macaca mulatta*) that were inoculated with 5 × 10^6^ plaque-forming units (PFU) of MPXV isolate Sierra Leone or Zaire 79 and fixed in 10% neutral-buffered formalin (NBF), were selected by the in-house pathologist. Samples included haired skin from 9 female and 2 male rhesus monkeys, with ages ranging from 5–14 years. The formalin fixation times of the skin ranged from 19–30 days (Table 2). Standard methods were used for paraffin-embedding, hematoxylin and eosin (H&E)-staining and chromogenic IHC-staining [38]. Subject description, MPXV isolates used and tissue fixation times are summarized in Table 2.

**Figure 1:**
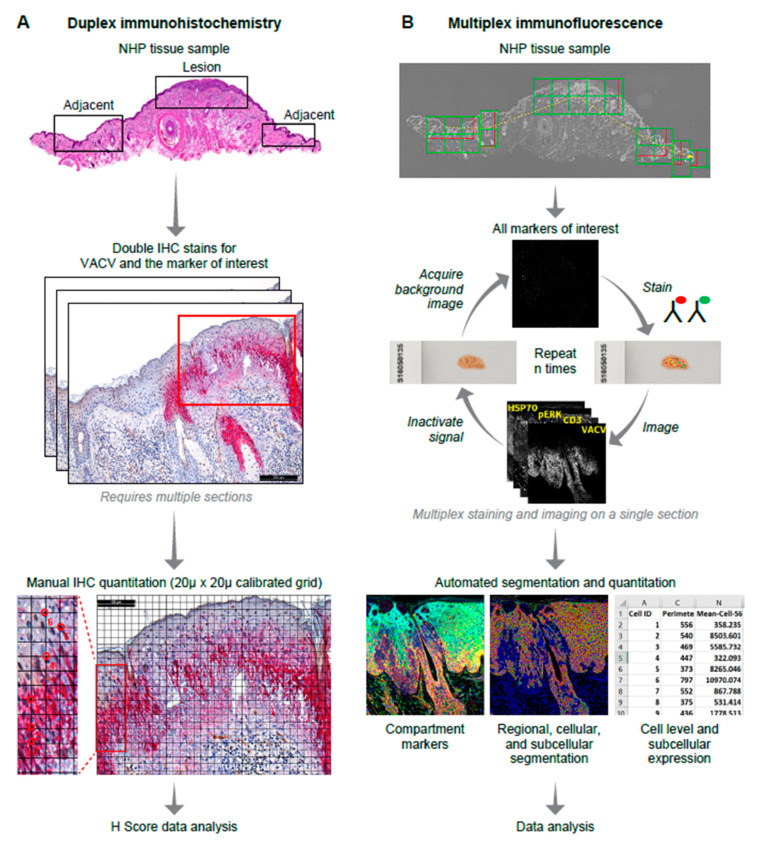
High-level immunohistochemistry (IHC) and multiplex immunofluorescence (MxIF) analysis workflows. Overall, workflows show staining processes and subsequent quantification approaches. (**A**) Double-stain IHC (one tissue section for each marker of interest and vaccinia virus (VACV)) followed by manual quantification; (**B**) MxIF workflow involves repeated staining, imaging and signal removal on a single tissue section followed by automated cellular/subcellular segmentation using compartment-specific markers and biomarker quantitation. NHP—nonhuman primate.

Four types of biomarkers were included in this study: viral protein markers, inflammatory cell cluster of differentiation markers, cellular stress markers invoked during the host epithelial cell response and mitogen-activated protein kinase markers (Table 1). In addition to the markers listed in Table 1, antibodies against CD8 (Dako M7103) and CD79a (Dako M7050 and Abcam AB3121) were also evaluated by chromogenic IHC and Cell DIVE MxIF. Using IHC, anti-CD8 antibody nonspecifically stained multiple cell types (including macrophages, small lymphocytes and neutrophils). Further, using double-stain IHC, anti-CD8 antibody also co-stained with anti-CD68 antibody. Similar nonspecific staining was found with the Cell DIVE method. Due to poor specificity, we opted to exclude CD8 from further analysis. The CD79 Dako M7050 antibody did not stain well using IHC, even after testing multiple antigen retrieval methods. Using Cell DIVE, this antibody also failed to stain in the test samples but stained weakly/specifically on the multi-tumor TMA. We hypothesized that epitope alteration by longer fixation may have been a contributory factor. On the other hand, the Abcam AB3121 CD79 antibody stained well by IHC in the test samples but failed using the Cell DIVE method. This discrepancy may be attributed to differences in antigen retrieval. As we were unable to do a head-to-head comparison, CD79 was excluded from further analysis. The orthopoxvirus antibodies, including those against cowpox virus (CPXV), MPXV, variola virus (VARV) and vaccinia virus (VACV), are cross-reactive [39,40], which allowed us to use an anti-VACV antibody (raised against vaccinia virus) as a surrogate marker to quantify MPXV in florid poxvirus skin lesions. Prior to multiplex staining and imaging, the antibody was compared against its isotype control on MPXV-infected tissue and was found to be specific. Additionally, staining was localized to the lesion clearly visible on virtual H&E (Figure 1). Regions of interest (ROIs), including MPXV-induced vesiculopustular lesions and 1–2 adjacent nonlesion areas, were marked by a pathologist for imaging and quantitation (see Figure 1A,B workflows). Similar ROIs were marked for both brightfield imaging (for chromogenic stains) and fluorescence imaging (for Cell DIVE analysis).

### 2.2. Antibody Conjugation and Validation

Previously validated standard IHC methods with secondary antibody detection were used for chromogenic staining. Cell DIVE requires a single two-step antigen retrieval process and staining using fluorescent dye conjugates of commercially available primary antibodies, for which a standardized antibody validation process has been developed (described in the supplementary material of Gerdes et al. [4]). This robust workflow is necessary to evaluate whether staining performance is similar to secondary antibody detection and demonstrates compatibility with a modified antigen retrieval method.

### 2.3. H-Score Analysis of Chromogenic IHC Stains

Double stains for VACV and the marker of interest were performed for each chromogenic IHC stain. H-score quantitation for each of the protein markers was performed by one in-house pathologist by counting a total of 100 cells using the predetermined ROIs from lesioned and adjacent MPXV–IHC-negative skin. The number of positively stained cells was measured as the percentage of the total numbers of counted cells and expressed as percent-positive cells. The staining intensity for each marker was visually scored 0–3 (0 = no staining, 1 = weak staining, 2 = moderate staining, 3 = strong staining) resulting in H-scores of percent-positive cells that ranged from 0 (no staining) to 300 (diffuse, intense staining) [41]. Manual cell counts were performed at 20× magnification.

To reduce visual bias during manual cells counts (H-scoring), a calibrated, computerized grid composed of squares measuring 20 µm across was applied to the ROIs using Image Pro Analyzer 7.0 software (Media Cybernetics, Rockville, MD, USA). Cells were counted from the left of the preselected ROIs and continued to the right following the horizontal lines of the computerized grid until a total count of 100 was reached. To further reduce visual counting bias, only those cells whose nuclei were overlaid by cross hatches of the computerized grid were counted (Figure 1A).

### 2.4. Multiplex Immunofluorescence Staining, Imaging and Image Processing

A high-level overview of the multiplex immunofluorescence (MxIF)-staining, imaging, image processing and data analysis workflow (Cell DIVE, Cytiva, Issaquah, WA, USA) is provided in Figure 1B [4]. Cell DIVE involves repeated cycles of staining, imaging and dye inactivation for 60 + biomarkers in a single FFPE tissue section [4]. Before undergoing multiplexed staining, each epitope is tested for potential sensitivity to the dye inactivation protocol (a mild chemical oxidation method). To date, we have found about 10% of epitopes to have signal reduction after 1–10 inactivation cycles (compared to a control slide not associated with an inactivation protocol), and those targets are stained earlier in the cycling process to mitigate against this effect [4]. In the current study, none of the biomarker targets degraded following dye inactivation and were positioned throughout the cycling process. The segmentation marker, ribosomal protein S6, was previously shown to be associated with signal degradation following dye inactivation and hence was placed in the first round along with VACV (in that case, primary and secondary antibodies were used for detection and those are typically positioned in the first round to avoid species cross-reactivity). We have also previously compared multiplexed versus singleplexed staining performance for a wide range of biomarkers and tissue types and have not found a difference in staining performance, indicating that steric hinderance does not seem to be an issue [4,9,10]. For this study, the archived samples were deparaffinized, hydrated and processed through a two-step antigen retrieval process [42] prior to blocking and staining with validated antibody conjugates (Table 1). All samples were batch-processed and batch-stained and underwent the same antibody staining sequence and antibody concentration (shown in Table 1).

Imaging was performed with Olympus IX-81 microscopes (Olympus Surgical & Industrial America, Orangeburg, NY, USA) outfitted with Cell DIVE image acquisition and image processing software for repetitive imaging of the same field-of-view (FOV). Microscopes were calibrated daily using dye-impregnated gels for illumination correction. DAPI (ThermoFisher Scientific, Waltham, MA, USA) signal from each round was used for registration of images (to the baseline round) prior to autofluorescence subtraction, cell segmentation and automated quantification of marker expression. Exposure times for imaging were predetermined for each biomarker based on a visual assessment of fluorescent intensity. Overexposure protection was activated by the Cell DIVE image acquisition software when fluorescence is stronger than expected. The fields of view (FOV) that were imaged in each sample are provided in Supplementary Table S1. Depending on sample dimensions, anywhere from 22–43 FOV were imaged per sample. For visual presentation and interpretation, all images for a given marker were adjusted to same window/level values in ImageJ (https://imagej.nih.gov/ij/).

### 2.5. Tissue, Single Cell and Subcellular Segmentation

Typically in the Cell DIVE image analysis process, Na^+^/K^+^-ATPase and/or cadherins are used for membrane segmentation of neoplastic epithelial tissues [4]. However, during analysis of the epithelium for this study, the staining patterns were not consistent across all epithelial cells in the sample, leading to under-segmentation of those cells. Therefore, to completely segment all available epithelial cells, a composite membrane stain was generated by empirically testing different combinations of Na^+^/K^+^-ATPase, pan-cytokeratin (panCK) and E-Cad images to arrive at the combination composite “stain” (panCK*(0.5 Na^+^/K^+^-ATPase + 0.5 E-Cad)). Whole cell and subcellular segmentation was performed as described previously by Gerdes et al. [4] using panCK (epithelial compartment), composite membrane marker described above (membrane compartment), RPS6 (cytoplasmic compartment) and DAPI (nuclear compartment).

To separate the stromal (or dermal) regions from epithelium, whole tissue masks were first generated to remove non-tissue regions from the edges of the tissue (i.e., glass at edge of tissue). A combination of tissue autofluorescence in the green fluorescent protein channel and DAPI was used to generate these tissue masks. Stromal regions were calculated by subtracting the epithelial regions defined by panCK from the whole tissue masks. Cellular biomarker data were quantified on a continuous scale.

A machine learning algorithm [43] developed at GE Research Center was used for binary classification of CD3^+^ and CD68^+^ cells. Individual cell types were quantified either as proportion of total cells in the stromal region, epithelium and whole image or number of cells per mm^2^.

### 2.6. Data Processing

DAPI signal registration (described above in Section 2.3) was used to generate a registration quality score ranging from 0–1 (0 for no registration and 1 for perfect registration) for each cell in each round (round level quality score), as well as an overall quality score (again from 0–1) for registration of cells through all rounds. These scores (per round or minimum across all rounds) are used to remove cells that may have shifted or are lost during multiplexing. This is important for cell level analysis of multiplexed data for which perfect or near-perfect cell registration is required for analysis of co-expressing markers. For this study, cells with a minimum quality score of 1 across all rounds were used (meaning only cells with perfect image registration across the 6 staining rounds were included in the analysis). Staining quality was assessed manually by visualizing staining patterns of individual markers across all samples.

**Figure 2:**
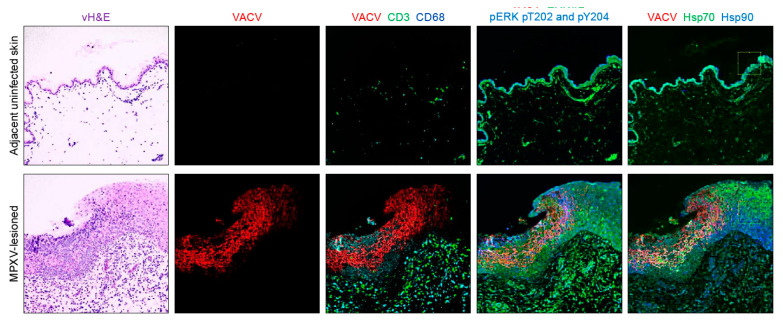
Examples of virtual hematoxylin and eosin (vH&E) and multiplexed staining by Cell DIVE of VACV, CD3, CD68, ERK1/2, ERK1/2 pT202_pY204, Hsp70 and Hsp90 in archived fixed monkeypox virus-induced skin lesions and in adjacent, nonlesion uninfected skin.

A “virtual” H&E (vH&E) was generated for each FOV (Figure 2). This virtual H&E presents the image as a H&E-like image based on pseudo-coloring of the grayscale DAPI and autofluorescence image to a purple/pink scheme. To verify consistent and correct epithelial and single cell segmentation, the segmented images (as described in 2.4) were visually assessed and compared with the corresponding virtual H&E, the original images of panCK, Na^+^/K^+^-ATPase and E-Cad, and the composite membrane mask (panCK*(0.5 Na^+^/K^+^-ATPase + 0.5 E-Cad). Images with failed segmentation (<2% of total images), due to weak staining or failed registration of one or more segmentation markers, were excluded from the analysis (Supplementary Table S1). Further data filtering included removal of non-cell objects created by over-segmentation of the image and/or segmentation of non-cellular regions. A “true cell” was required to have a minimum of 10 pixels per subcellular compartment (nucleus, membrane and cytoplasm) and no more than two nuclei. Cells not meeting those criteria were removed from data analysis.

Since illumination across the FOV was nonuniform, images underwent field flattening using in-house-prepared (GE Research) dye-impregnated gels. Calibration standards were also imaged every day prior to imaging and the resulting calibration files were used for image processing and quantification at cellular and subcellular level. All biomarker intensities on all the slides were adjusted to a common exposure time per channel. The quality-checked single cell biomarker data were log_2_-transformed, normalized to the median intensity for each biomarker and cellular intensities were averaged at region-level (lesion and adjacent uninfected skin).

### 2.7. Statistical Analysis

Biomarker data for both isolates (Zaire 79 and Sierra Leone) were first evaluated separately and since the results for each group were similar, the datasets from each group were combined. Using the H-score biomarker data, paired t-tests were performed to compare lesion versus adjacent nonlesion regions for the chromogenic IHC. An adjustment for multiple comparisons was not made.

For the MxIF single cell data, the cell expression difference between lesion and adjacent nonlesion tissue regions for each marker per animal was calculated (depending on sample dimensions, between 22–43 FOV were imaged within each region (supplementary Table S1) and each FOV comprised of several thousand cells). One sample t-test was used to test the null hypothesis that the expression difference between lesion and adjacent nonlesion regions was zero. The raw *p*-values from the one sample t-test were generated. Since multiple markers were tested simultaneously, *q*-values (false discovery rate) correcting for the multiplicity were estimated using Benjamini–Hochberg procedure (implemented in R library qvalue (R Core Team (2013); R: a language and environment for statistical computing. R Foundation for Statistical Computing, Vienna, Austria. URL http://www.R-project.org)).

## 3. Results

As described earlier, four types of biomarkers were included in this study comparing IHC-based H-scores and automated cell level quantitation: viral protein markers, inflammatory cell cluster of differentiation markers, cellular stress markers invoked during the host epithelial cell response and mitogen-activated protein kinase markers (Table 1). For automated cell-level biomarker quantitation, panCK, Na^+^/K^+^-ATPase, E-Cad, RPS6, nuclear DNA (with DAPI)), were also stained, but not included in the statistical analysis. MPXV protein presence (using the MPXV cross-reactive anti-VACV antibody)) was significantly higher in infected regions vs adjacent nonlesion regions (*p* < 0.0001). No difference in MPXV distribution was found between the either MPXV isolate using IHC or Cell DIVE. Figure 2 shows two example image sets from the adjacent nonlesion skin and MPXV-lesioned skin and biomarker image overlays for each.

### 3.1. CD68

A statistically significant increase in CD68^+^ cell numbers (*p* = 0.02) in epithelium was measured using H-scoring in MPXV-lesioned skin when compared to adjacent, nonlesion skin regions. This increase was also observed in the proportion of CD68^+^ cells in the MPXV-lesioned regions using Cell DIVE (epithelium (*p* = 0.017), dermis (*p* = 0.006) layers and overall (*p* = 0.003)) (Table 3).

### 3.2. CD3

H-scoring showed an increase in CD3^+^ cell numbers in epithelium of lesioned skin when compared to adjacent, nonlesion skin, but did not reach significance (*p* = 0.062). Cell DIVE showed a significant increase in CD3+ cell numbers in MPXV-lesioned skin compared to adjacent nonlesion skin (overall (*p* = 0.018) and dermal compartment (*p* = 0.034). No change was apparent in the epithelial compartment.

### 3.3. Heat-Shock Proteins 70 and 90

**Figure 3:**
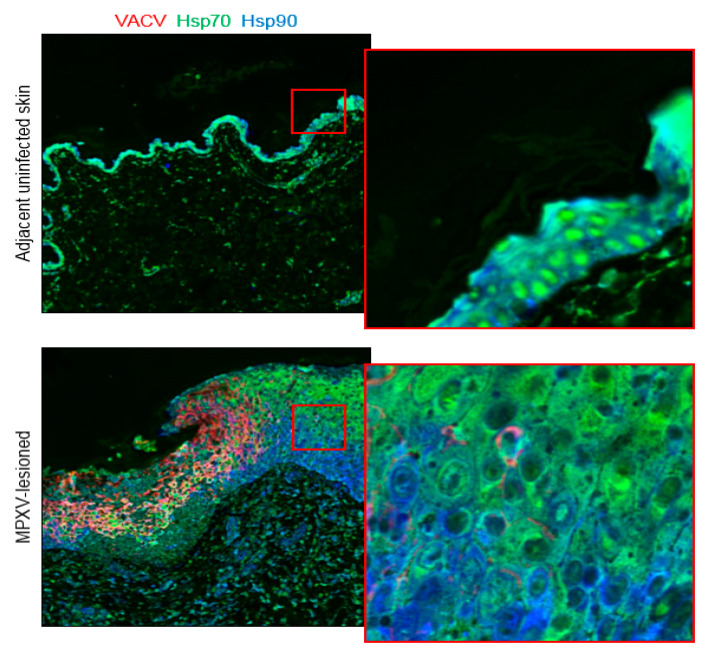
Differential subcellular localization of Hsp70 in adjacent nonlesion vs infected skin regions. In adjacent nonlesion regions, a strong nuclear signal for Hsp70 is observed, whereas in the infected lesion area it is mainly cytoplasmic. For Hsp90, no significant difference in expression was detected in the cytoplasm or nuclei of lesioned skin using IHC or Cell DIVE.

Using H-scoring, no significant difference in Hsp70-staining was detected in the cytoplasm (*p* = 0.294) or nuclei (*p* = 0.139) in MPXV-lesioned regions compared to adjacent, nonlesion skin regions. In contrast, Cell DIVE showed a significant decrease in Hsp70 expression in MPXV-lesioned skin, both in the cytoplasm (*p* = 0.002) and nuclei (*p* = 0.0001) (Table 3). Interestingly, using Cell DIVE a higher cytoplasmic to nuclear ratio of Hsp70 expression was observed in the MPXV-infected region (*p* = 5.78 × 10^−6^) compared to nonlesion uninfected regions. This result was reflected in Figure 3, where the infected lesion is characterized by a diffuse cytoplasmic staining (with diminished nuclear staining) with a strong nuclear staining in the adjacent nonlesioned skin region. No significant difference in Hsp90-staining was detected using H-scoring in the cytoplasm (*p* = 0.169) or nuclei (*p* = 0.573) in lesioned skin compared to adjacent, nonlesion skin. Similarly, no significant difference in Hsp90 expression was detected with Cell DIVE in the cytoplasm (*p* = 0.325) or nuclei (*p* = 0.144).

### 3.4. Unphosphorylated Extracellular Signal-Regulated Kinases 1 and 2

A significant decrease in unphosphorylated ERK1/2-staining within the cytoplasm (*p* = 0.013) was measured in MPXV-lesioned skin when compared to adjacent, nonlesion skin using H-scoring. No difference in nuclear staining of ERK1/2-staining was measured in MPXV-lesioned skin compared to adjacent nonlesion skin (*p* = 0.23). Similarly, using Cell DIVE, ERK1/2 expression was significantly lower in lesioned skin for both cytoplasm (*p* = 2.59 × 10^−4^) and the nuclei (*p* = 4.62 × 10^−4^) (Table 3) compared to adjacent nonlesion regions.

### 3.5. Phosphorylated Extracellular Signal-Regulated Kinases 1 and 2

Using H-scoring, no significant difference in ERK1/2 pT202_pY204 in the cytoplasm (*p* = 0.883) of MPXV-lesioned skin compared to adjacent, nonlesion skin was found. However, a significant increase in phosphorylated ERK1/2 pT202_pY204-staining was found in MPXV-lesioned skin in the nuclei (*p* = 0.004). In contrast to the H-scores, using Cell DIVE, ERK1/2 pT202_pY204 expression was found to be lower in lesioned skin regions compared to adjacent nonlesion regions in both the cytoplasm (*p* = 1.96 × 10^−3^) and nuclei (*p* = 2.24 × 10^−4^) (Table 3 and example images in supplementary Figure S1).

## 4. Discussion

In this study, we used semiquantitative manual H-scoring and automated single cell analysis using Cell DIVE multiplexed imaging workflow to measure the expression of two cluster of differentiation markers (CD68 (macrophages) and CD3 (T-cells)), two heat shock protein markers (Hsp70 and Hsp90) and a mitogen-activated protein kinase (ERK1/2) and phosphorylated ERK1/2 (ERK1/2 pT202_pY204) in MPXV-infected rhesus monkey skin compared to adjacent nonlesion skin. The goal of this study was to determine whether automated single cell quantitation of these biomarkers was comparable to manual H-scoring in complex inflammatory skin lesions. The Cell DIVE segmentation and quantitation algorithms generate both continuous expression values at the cell and subcellular levels, as well as binary classification of cells as positive or negative; the latter is generated using a machine-learning based approach. In this study, we generated continuous subcellular data for the Hsp and ERK markers and binary data for CD3-and CD68-positive cells. Although we did not generate “H-score” measurements from the multiplexed cellular data, in principal it is possible by manually or computationally applying thresholds (low, medium, strong) to the biomarker intensity data and calculating proportions of cells within each staining intensity category. Increases in both dermal and intra-epidermal macrophages and CD3^+^ T-cells were expected in lesioned skin when compared to adjacent, MPXV-negative skin [21,24]. A statistically significant increase in CD68 macrophage numbers was measured using both methods. Although T-cell numbers were also expected to be higher in lesioned skin compared to adjacent uninfected skin, they did not reach statistical significance with manual H-scoring [21,24]. Cell DIVE did show a statistically significant increase in lesioned skin, but only in dermis, not epidermis. These results may be due to the both the small sample size used for this proof-of-concept study and the larger number of cells that are quantified using Cell DIVE compared to the H-score method [43].

VACV infection of macrophages in vitro and in mouse splenocytes in vivo causes marked increases in Hsp70 concentrations [28,29,44]. However, constitutive and ubiquitous expression of Hps70 in the skin leads to dense and diffuse IHC-staining that is difficult to quantify visually. We did not detect differences in either Hsp70 or Hsp90-staining using H-scoring in MPXV-lesioned skin compared to adjacent MPXV-negative skin. With Cell DIVE, total cellular expression of Hsp70 was significantly lower in the MPXV-lesioned skin compared to the adjacent nonlesion, uninfected skin region and compartmentalization of expression shifted from nuclei to cytoplasm (Figure 3). In addition, expression appeared more intense in the more superficial regions of the hyperplastic epidermis compared to the stratum basale and spinosum cell layers, (although not in all examined samples). This finding contradicts previously reported results for diverse orthopoxviruses, including MPXV [25,26,27,28,29,44], but may reflect superior cell segmentation capabilities of Cell DIVE compared to IHC. Hsp90, like Hsp70, has a dense, diffuse chromogenic IHC-staining pattern, making detection of changes in both staining intensity and cytoplasmic localization challenging. No changes in Hsp90-staining using H-scoring or Cell DIVE were detected. Similar to IHC, Cell DIVE revealed a diffuse signal throughout the cytoplasm of the epidermis (Figure 3).

Phosphorylation of ERK1/2 is required for VACV replication [32]. Increases in ERK1/2 pT202_pY204 expression and translocation into the nucleus were previously documented in orthopoxvirus-infected cells [32]. Our findings in lesioned versus adjacent MPXV-negative skin using H-scoring confirm these events for MPXV. However, automated quantification with Cell DIVE suggests the opposite trend where we found a decrease in pERK in lesions compared to the adjacent, nonlesion skin, which may be related to how cells are segmented using the automated method (discussed below).

**Figure 4:**
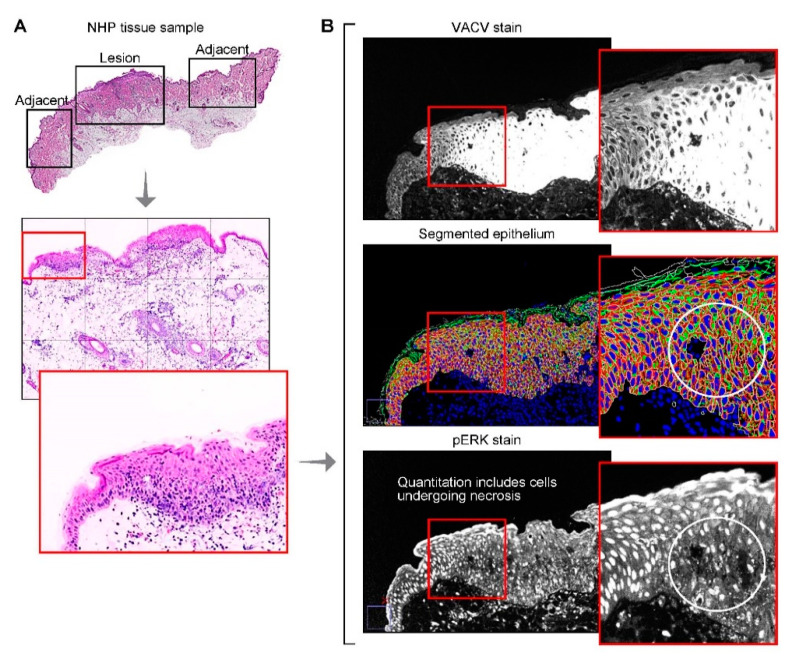
Multiplex immunofluorescence, segmentation and quantification. A potential cause of the discrepancy between semiquantitative manual IHC score and automated quantification of pERK1/2 is the inclusion of successfully segmented, but nonviable cells when using Cell DIVE. (**A**) H&E of monkeypox virus-lesioned skin and adjacent, uninfected skin annotated by a pathologist (top); H&E of the lesion included in the automated quantification (middle); H&E of a single field of view within the lesioned skin (bottom). (**B**) MPXV-staining in the field of view shown in (A) (top); Automated cellular/subcellular segmentation using compartment and region-specific cell markers (middle, bottom); ERK1/2 pT202_pY204-staining indicated loss of ERK1/2 pT202_pY204 in nonviable cells (bottom).

One limitation of the automated single cell segmentation approach is that all cells and subcellular compartments (e.g., nucleus, cytoplasm and membrane) are counted and included in analysis, independent of cell viability. During manual H-scoring, the pathologist discounts cells that are obviously nonviable. Additionally, any epithelial cell that stains positively for pan-cytokeratin is included in the epithelial cell biomarker analysis, irrespective of its location (epidermis or dermis (e.g., hair follicles)). Non-viable cells can be removed at the data analysis stage using Cell DIVE, based on cell size or absence of staining in one or more of subcellular regions (Figure 4), but removal of hair follicles may require manual exclusion of regions containing these structures. The inclusion of additional markers, such as annexin V to facilitate the identification of non-viable or apoptotic cells, stem cell markers to identify hair follicle cells and machine-learning-based segmentation should be considered to improve Cell DIVE quantitative performance and to clarify contradicting results between traditional and novel methods. It is also relevant to point out that although adjacent nonlesion regions did not show evidence of infection, expected cellular changes may not solely be limited to the infected regions. Altered cellular responses in adjacent non-infected regions may occur, but their identification will require additional spatial analyses.

Interestingly, we also observed that the two markers routinely used for Cell DIVE cell segmentation of the epithelium in human tumors (Na^+^/K^+^-ATPase and cadherins) were not uniformly expressed in rhesus monkey epithelium and appeared to be mutually exclusive (data not shown). In addition, stromal regions were stained with E-Cad, forcing us to develop an empirically derived combination of markers to cleanly and completely segment the epithelium. These issues highlight the need for the additional development of animal species-specific segmentation markers to facilitate the automated segmentation and quantification of cells using the Cell DIVE platform.

Protein marker quantification using chromogenic IHC required labor-intensive, double immunohistochemical staining for MPXV and the marker of interest on individual serial tissue sections followed by manual quantification of each double stain. To draw conclusions using multiple markers, the lesion of interest had to be both large and uniform. In contrast, using Cell DIVE, all six protein markers were analyzed within the same tissue section, providing sample consistency, the ability to identify individual cell types and the colocalization and subcellular localization of markers within those cell types. Importantly, the two quantification methods for markers of inflammation in this study were in overall agreement. This suggests that Cell DIVE may be used in place of manual H-scoring to quantify inflammatory cells, even if tissue samples were formaldehyde-fixed for up to 30 days. As noted earlier, for some markers, the inclusion of non-viable cells by the automated cell segmentation process may result in discrepancies, as was found for pERK. Although manual H-scoring is considered the “gold-standard” quantification method [45], confirmation of either the H-scores or Cell DIVE findings by measuring tissue protein concentrations of pERK was not possible in this retrospective study. To improve accuracy, further development of Cell DIVE for the analysis of tissues with extensive degeneration and necrosis should include the addition of cell viability markers and markers of cell death.

Although most markers stained well in this study, CD8 and CD79 did not and hence were excluded. The discrepancies observed for CD8- and CD79-staining could be attributed to tissue fixation and/or antigen retrieval differences in the methods. For CD8, it appeared to be an issue of poor antibody specificity, regardless of conditions or method. For CD79, the Dako antibody did not work well for either IHC or Cell DIVE using the test samples, but specific, but weak staining for CD79 was observed in the multi-tumor TMA slide using Cell DIVE. The Abcam CD79 antibody worked well for IHC on test samples but failed using Cell DIVE (so was attributed to antigen retrieval differences). In summary, it is important to establish a standardized antibody validation workflow to test multiple antibody clones and to include reliable positive and negative controls at standard fixation and prolonged fixation to rule in or out fixation effects or other method differences.

As mentioned in the methods section, an important feature of the Cell DIVE workflow is the ability to exclude cells that do not align or register well to the baseline image. Having perfect or near-perfect cell registration is especially important for analysis of co-expressing marker and co-locating markers. Although we did not quantify how many removed cells were orthopoxvirus-infected, thousands of cells were included in the final analysis and the risk of bias was likely to be extremely low. Although removing debris and cell-like material provides an enriched and cleaner cellular dataset, however depending on the scientific or mechanistic question, it may be useful in some instances to retain this information (e.g., for quantifying the spatial cellular response near regions of necrosis).

In conclusion, we have directly compared multiplexed immunofluorescence imaging and automated single cell analysis of a single tissue section with standard chromogenic staining and H-scoring on multiple tissue sections. Overall, the methods yielded directionally similar results and differences in levels of significance and/or direction (ERK1/2 pT202_pY204) may be attributed to the unsupervised/indiscriminate analysis of thousands of cells by Cell DIVE, compared to more standardized H-score analysis of a smaller number of cells in defined, viable regions. Future advances in machine learning image analysis and inclusion of viability markers should narrow those differences. Multiplexed imaging provides other advantages for BSL-3 and BSL-4 viral pathology research (e.g., with Ebola or Lassa viruses) because it is tissue-and time-sparing, allows for the colocalization of a multitude of markers within a single tissue section and potentially provides new insights into infection mechanisms. Since potentially dozens of biomarkers can be analyzed in a single tissue section, future research may include other mechanistic biomarkers, spatial cell analysis and identification of immune response/suppression mechanisms.

## Figures and Tables

**Table 1:** Orthopoxvirus-associated protein, biomarkers and image segmentation markers used in this study.

**Marker**	**Abbreviation**	**Antibody Clone**	**Staining Round**	**Stain Concentration (µg/mL) for Cell DIVE**	**Target**	**Cellular Location**	Source */Catalog Number
Vaccinia virus	VACV	8115	1	2	MPXV-infected cells	Cytoplasm, membrane	Santa Cruz/sc-58,210
Cluster of differentiation marker 68	CD68	KP1	5	5	Monocytes/macrophages	Cytoplasm	Thermo Fisher/MS-397-PABX
Cluster of differentiation marker 3	CD3	F7.2.38	6	5	T cells	Membrane	Dako/M7254
Heat shock protein 70	Hsp70	EP1007Y	5	5	Epithelial cells	Cytoplasm and nucleus	Epitomics/1776
Heat shock protein 90	Hsp90	C45G5	4	10	Epithelial cells	Cytoplasm and nucleus	Cell Signaling/4877
Extracellular signal-regulated kinases 1/2	ERK1/2	137F5	4	5	Epithelial cells	Cytoplasm	Cell Signaling/4695
Phosphorylated extracellular signal-regulated kinases 1/2 pT202_pY204	ERK1/2 pT202_pY204	20G11	3	5	Epithelial cells	Cytoplasm and nucleus	Cell Signaling/4376
**Segmentation Markers for Cell DIVE**	**Abbreviation**	**Antibody Clone**	**Staining Round**	**Staining Concentration (µg/mL)**	**Target**	**Cellular Location**	**Source/Catalog Number**
4′,6′-diamidino-2-phenylindole	DAPI	NA	all	10,000	All cells	Nucleus	Thermo Fisher/D3571
Ribosomal protein S6	RPS6	5G10	1	5	All cells	Cytoplasm	Cell Signaling/2217
E-cadherin	E-Cad	24E10	2	5	Epithelial membranes	Membrane	Cell Signaling/3195
Pan-cytokeratin (Epithelial marker)	panCK, a cocktail (ratio 1:2) of AE1 and PCK26	AE1/PCK26	2	2.5	Epithelial cells	Cytoplasm	eBioscience/14-9001 & Sigma/C1801
Sodium/potassium ATPase, alpha-1	Na^+^/K^+^-ATPase	EP1845Y	3	5	Epithelial cells	Membranes	Epitomics/2047

All Cell DIVE antibody incubations were conducted at ambient temperature for 1 h. * Santa Cruz Biotechnology, Inc., 10410 Finnell Street, Dallas, TX 75220, USA; Thermo Fisher Scientific, 168 Third Avenue, Waltham, MA 02451, USA; Dako (Agilent) 5301 Stevens Creek Blvd., Santa Clara, CA 95051, USA; Epitomics (Abcam), 1 Kendall Square, Suite B2304, Cambridge, MA 02139, USA; Cell Signaling Technology, 3 Trask Lane, Denvers, MA 01923, USA; eBioscience Inc., 10255 Science Center Drive, San Diego, CA 92121, USA.

**Table 2:** Monkeypox virus-positive haired skin sample collection from rhesus monkeys infected at a dose of 5 × 10^6^ PFU.

Sample No.	Sex	Age at Necropsy	MPXV Isolate	Days Post-inoculation to Necropsy	10% NBF Fixation Time (d)
1	F	9	Zaire 79	9	29
2	F	10	Zaire 79	8	30
3	F	14	Sierra Leone	9	19
4	F	13	Zaire 79	9	29
5	M	4	Zaire 79	8	30
6	F	13	Zaire 79	8	30
7	F	10	Sierra Leone	7	21
8	M	9	Sierra Leone	8	20
9	F	9	Sierra Leone	9	19
10	F	14	Sierra Leone	9	19
11	F	5	Sierra Leone	8	20

MPXV—monkeypox; NBF—neutral-buffered formalin.

**Table 3:** Biomarkers differentially expressed between monkeypox virus-infected and adjacent nonlesion skin regions using IHC and Cell DIVE.

	H-Score *N* = 11				Cell DIVE *N* = 11			
Adjacent VACV (−) Nonlesion Skin Compared to VACV (+) Lesioned Skin (*t*-Tests)	Difference in Mean	Std of Difference	*t*-Value	*p*-Value	Difference in Mean *	Std of Difference	*t*-Value	*p*-Value
CD68 Dermis	−0.025	0.286	−0.087	0.78	−0.015	0.005	−3.339	0.006
CD68 Epithelium	−0.368	0.44	0.877	0.02	−0.011	0.004	−2.801	0.017
CD3 Dermis	0.17	0.509	0.334	0.292	−0.015	0.006	−2.416	0.034
CD3 Epithelium	−0.53	0.838	−0.632	0.062	0.005	0.003	1.6561	0.126
Proportion of CD3 cells in the epithelium and stroma	N/A	N/A	N/A	N/A	−0.015	0.005	−2.770	0.018
Proportion of CD68 cells in the epithelium and stroma	N/A	N/A	N/A	N/A	−0.0168	0.005	−3.671	0.003
Hsp70 epithelium cytoplasm	0.104	0.311	0.334	0.294	0.314	0.080	3.916	0.002
Hsp70 epithelium nucleus	0.303	0.625	0.484	0.139	0.502	0.087	5.799	0.0001
Hsp90 epithelium cytoplasm	−0.106	0.21	−0.505	0.169	0.109	0.106	1.030	0.325
Hsp90 epithelium nucleus	0.022	0.199	0.111	0.573	0.152	0.097	1.573	0.144
Unphosphorylated ERK1/2 epithelium cytoplasm	0.295	0.324	0.910	0.013	0.377	0.071	5.284	0.0003
Unphosphorylated ERK1/2 epithelium nucleus	0.133	0.347	0.383	0.234	0.395	0.080	4.913	0.0005
Phosphorylated ERK1/2 pT202_pY204 epithelium cytoplasm	0.01	0.219	0.046	0.833	0.482	0.120	4.036	0.002
Phosphorylated ERK1/2 pT202_pY204 epithelium nucleus	−0.189	0.17	1.11	0.004	0.604	0.112	5.379	0.0002

* The difference in log_2_ protein expression values with the exception of CD3 and CD68. CD3 and CD68 on Cell DIVE is the difference in proportion of positive cells in the dermis and epidermis. Std: standard deviation.

## Supplementary Materials

The following are available online at https://www.mdpi.com/1999-4915/12/8/787/s1, Figure S1: Examples of ERK1/2 pT202_pY204 staining by IHC and MxIF. Table S1: FOV Annotations with Segmentation Review.

## Disclaimer

The views and conclusions contained in this document are those of the authors and should not be interpreted as necessarily representing the official policies, either expressed or implied, of the US Department of Health and Human Services or of the institutions and companies affiliated with the authors. In no event shall any of these entities have any responsibility or liability for any use, misuse, inability to use or reliance upon the information contained herein. The US Department of Health and Human Services does not endorse any products or commercial services mentioned in this publication.
